# Supplementary material for: Peritoneal dialysis after failed kidney allograft: Comparing patients with and without pd before transplant
Source: PLoS One. 2023 Jul 18;18(7):e0284152. doi: 10.1371/journal.pone.0284152 (PMC10353794; doi:10.1371/journal.pone.0284152)
Supplement: Attachment — Submitted filename: PDFTx PLoS reply.docx [file pone.0284152.s002.docx]

15 February 2023

Editor-in-Chief

PLoS One

Sir,

Re: ‘Peritoneal Dialysis after Failed Kidney Allograft: Comparing Patients With and Without PD Before Transplant’ (PONE-D-22-34904)

Thanks for your letter on 23 January. The following revisions have been made according to your recommendations:

**Reviewer 1**

**The summary is a fair and effective summary of the main text. However, I suggest that the conclusion be the same as the conclusion in the main text. I mean, in the abstract, the authors conclude that all patients have increased patency if they had PD before transplantation, whereas in the conclusion of the article, they are less formal and say that this is favored by a history of PD-related peritonitis. The conclusion of the abstract should be as compelling as the conclusion of the article.**

Page 26, last paragraph: As suggested, we revised the wordings of the concluding paragraph and make it in line with the Abstract.

**In the last paragraph of the "study end point" section: patient survival is correctly defined, but for technical survival it is written "Technical survival was defined as the patient remaining alive and on PD". There are two different ways of calculating technical survival; it was not clear whether the definition of technique failure included transfer to HD, death or transplantation or whether death and transplantation were censored.**

Page 7, paragraph 1, line 3-5: As suggested, we clarify the definition of events for technique survival analysis.

**In the paragraph "Patient and Technique Survival," the authors explain that "in the group of PD patients after failed transplantation, 14 patients received a second kidney transplant." Was the number of secondary renal transplants the same in the subgroup of patients with PD before transplantation compared with those without a history of PD before transplantation, because second transplants may be considered competitive events that could alter the estimate of technical survival**

Page 7, paragraph 1, line 4-5; page 15, paragraph 1, line 4: We clarify the number of patients who had a second transplant in the subgroup with PD before transplant. We also clarify that second transplantation was treated as a competing event for technique survival analysis.

**Tables and figures are well documented in their legend and support the understanding of the main text. In the legend of figure 1, explain the technique survival (was failure censored on death and transplant or only transplant ?)**

Figure 1 legend: We elaborate on the definition of events for patient and technique survival as suggested.

**Reviewer 2**

**Please indicate when D/P4 was measured in the first PD in patients with PD before transplant. Is it 1 to 2 months after the patients were stable on PD, similar to the timing of PD after graft failure?**

Page 6, paragraph 1, line 8: We clarify that for patients with PD before transplant, the original PET was performed one to two months after they were first started on PD.

**Please indicate when Kt/V, residual GFR, NPNA, and FEBM were measured.**

Page 5, last line; page 6, line 1: We clarify that Kt/V, residual GFR, NPNA, and FEBM were measured 1 to 2 months after the patients were stable on PD.

**In Table 4, please indicate in the title that it is a summary of the comparison between transplant and no transplant. Also, please correct the "worse" so that it is clear which is worse, transplant or no transplant.**

Table 5: The title is revised as suggested. We also add a footnote to explain the meaning of “worse”.

**In line 12 of the Discussion 2nd paragraph, you indicate that reference 5 is not a matched study. However, actually reference 5 shows a matched cohort study. Please correct this point.**

Page 21, last 2 lines: The citation is updated. We are sorry for the mistake.

**In Tables 1 and 2, for covariates that are clearly not normally distributed such as Charlson's score and duration of PD before transplant, we think the Mann-Whitney test should be used instead of Student's t test. Similarly, when comparing nonparametric data between previous PD and no previous, the Wilcoxon signed-rank test should be used.**

Table 1, 2, and 3: We revised the analysis for Charlson’s score, and residual GFR by non-parametric test as suggested. The data between groups with and without previous PD were also compared by non-parametric test.

**Please make a list of causes of death and compare both groups if possible.**

Table 4: The cause of death is summarized in a new table as recommended.

**Please validate whether transplantation affects patient and technical survival using a multivariate-adjusted survival analysis in a total cohort combining both groups. Similarly, please validate whether previous PD before transplant affects outcome by adding previous PD as a covariate.**

Page 15, last 3 lines: Because of the small number of events and insignificant difference in the univariate analysis, further multi-variable Cox survival analysis was not performed.

**Despite being matched by DM status, the ratio of diabetic kidney disease is diverging between the control and all case group. Please describe the reason for this.**

Page 26, paragraph 1, line 3-6: We clarify that we matched the case and control groups by diabetic status, but it turns out that more patients in the control group had diabetic kidney disease as the underlying cause of kidney failure. The potential selection bias is elaborated in the discussion.

**In Table 1, please indicate the number of men for gender, and indicate % in parentheses.**

Table 1: We describe the number of men for gender as suggested.

**In Table 3, “duration of transplant before PD” should be described as “duration of transplant”. It can be confusing for patients who have had PD before and after transplant.**

Table 3: The phrase is revised to “duration of transplant” as suggested.

**Please describe the unit of duration of hospitalization in Fig 2 in A and C. Is it a month?**

Figure 2: We updated the unit of duration of hospitalization in the figure axis.

**The reference numbers in Table 4 do not exist in the manuscript. Please correct this point.**

Table 5: We updated the reference number in the table. We are sorry for the mistake in the previous manuscript.

We would like to resubmit the manuscript, with the changes in red, for consideration of publication in the PLoS One. Thank you for reviewing our article and we look forward to hearing your favorable reply.

Yours faithfully,

CC Szeto

For N Tian, H Meng, WWS Fung, JKC Ng, GCK Chan, VWK Kwong, WF Pang, KM Chow, and PKT Li
