# Supplementary material for: Peritoneal dialysis after failed kidney allograft: Comparing patients with and without pd before transplant
Source: PLoS One. 2023 Jul 18;18(7):e0284152. doi: 10.1371/journal.pone.0284152 (PMC10353794; doi:10.1371/journal.pone.0284152)
Supplement: Attachment — Submitted filename: PDFTx PLoS reply c2.docx [file pone.0284152.s003.docx]

14 March 2023

Editor-in-Chief

PLoS One

Sir,

Re: ‘Peritoneal Dialysis after Failed Kidney Allograft: Comparing Patients With and Without PD Before Transplant’ (PONE-D-22-34904R1)

Thanks for your letter on 7^th^ March. The following revisions have been made according to your recommendations:

**Reviewer 2**

**In Table 4, the total of the number of PD and no PD before transplantation does not accord with the number of all cases.**

Table 4: The numbers are checked and one typo rectified. We are sorry for the mistake.

**In Fig 2 B and D, please change the unit to “days/patient-year”.**

Figure 2B and 2D: The label of the y-axis is changed as suggested.

In addition, the information of reference #21 is updated.

We would like to resubmit the manuscript, with the changes in red, for consideration of publication in the PLoS One. Thank you for reviewing our article and we look forward to hearing your favorable reply.

Yours faithfully,

CC Szeto

For N Tian, H Meng, WWS Fung, JKC Ng, GCK Chan, VWK Kwong, WF Pang, KM Chow, and PKT Li
